# Self-care conditioning factors in women and men with urinary incontinence and Human T-Lymphotropic Virus Type 1

**DOI:** 10.1590/1980-220X-REEUSP-2024-0086en

**Published:** 2025-01-27

**Authors:** Rayssa Fagundes Batista Paranhos, Juliana Bezerra do Amaral, Rose Ana Rios David, Anderson Reis de Sousa, Oscar Javier Vergara Escobar, Tatiane Assone dos Santos, Jeferson Moreira dos Santos, Rafael Costa Fernandes

**Affiliations:** 1 Universidade Federal da Bahia, Escola de Enfermagem, Programa de Pós-Graduação em Enfermagem e Saúde, Salvador, BA, Brasil.; 2 Pontificia Universidad Javeriana Sede Bogotá, Bogotá, Colômbia.; 3 Universidade de São Paulo, Departamento de Medicina Legal, Bioética, Medicina do Trabalho e Medicina Física e Reabilitação, São Paulo, SP, Brasil.; 4 Universidade Federal da Bahia, Escola de Enfermagem, Salvador, BA, Brasil.

**Keywords:** Self Care, Women's Health, Men's Health, Human T-lymphotropic virus 1, Urinary Incontinence, Autocuidado, Salud de la Mujer, Salud del Hombre, Virus Linfotrópico T Tipo 1 Humano, Incontinencia Urinaria

## Abstract

**Objective:**

To analyze self-care conditioning factors in women and men with urinary incontinence symptoms living with the Human T-Lymphotropic Virus Type 1.

**Method:**

Qualitative study, based on the pragmatic phase of Praxis Model for Technology Development. Twelve women and five adult men living with Human T-Lymphotropic Virus Type 1, residing in Bahia, Brazil, participated in the study. A structured instrument and focus group were applied to collect data. The empirical material was subjected to Thematic Content Analysis, using the *software* WebQDA and the Self-Care/Self-Care Deficit Theory was used.

**Results:**

Facilitating conditioning factors were used by women and men as strategies for performing self-care, and hindering conditioning factors prevented or disturbed, but opened paths to direct the nurse's performance to address self-care deficits.

**Conclusion:**

Self-care requirements were impacted by the hindering conditioning factors; the facilitating ones allowed self-care, providing technology in nursing/health.

## RESUMEN

**Objetivo:**

Analisar los fatores condicionantes para el autocuidado en mujeres y hombres con síntomas de incontinencia urinaria y Virus linfotrópico T humano tipo 1.

**Método:**

Estudio cualitativo, guiado por la fase Pragmática del Modelo Práctico para el Desarrollo Tecnológico. Participaron 12 mujeres y cinco hombres adultos viviendo con Virus linfotrópico T humano tipo 1, residentes de Bahía, Brasil. Para la recolección de datos se aplicó un instrumento estructurado y un grupo focal. El material empírico fue sometido a Análisis de Contenido Temático, utilizando el *software* WebQDA y la Teoría del Autocuidado/Déficit de Autocuidado.

**Resultados:**

Los condicionantes facilitadoras fueron utilizadas por mujeres y hombres como estrategias para realizar el autocuidado y los condicionantes obstaculizadoras impidieron o dificultaron, pero abrieron caminos para orientar las acciones del enfermero para superar los déficits de autocuidado.

**Conclusión:**

Los requisitos de autocuidado se vieron afectados por los factores condicionantes obstaculizadores; los facilitadores permitieron el autocuidado y brindaron oportunidades para la enfermería y la tecnología de la salud.

## INTRODUCTION

Human T-lymphotropic virus type 1 (HTLV 1) is transmitted through blood, vertical, and sexual contamination. Around 5 to 10% of the world’s population has the virus, with it being prevalent in Brazil in around 0.5 to 1% of the population^(1,2)^, and in some Brazilian states, such as Bahia, reaching 1.76% of the population^(1)^. There are four subtypes of the virus and type 1 is the one causing myelopathy, leading to neurological complications such as HTLV-1 Associated Myelopathy (HAM), progressing to neurological dysfunction of the lower urinary tract (LUTND). The latter is also known as neurogenic bladder, which may present symptoms of urinary incontinence (UI), overactive bladder, incomplete bladder emptying and urinary retention, leading to the need for clean intermittent bladder catheterization^(1,3)^. This study focuses on the symptoms of UI, which are broad and generic in people living with HTLV 1, being different from other urinary losses, since it can present several isolated or associated symptoms, making diagnosis and adequate management difficult, consequently requiring multiple therapeutic approaches.

The constant need to know how to deal with chronic health problems changes the individuals’ view of themselves, requiring them to take action to take care of themselves^(4)^. In this regard, daily activities initiated and carried out for oneself with the purpose of maintaining life, health and well-being, pass through the perspective of self-care. These activities are learned according to cognitive knowledge, beliefs, values, habits, and practices exercised throughout life and which can be strengthened or impaired, depending on the professional guidance received and the collective culture in which the person finds him/herself inserted^(4,5,6)^.

Self-care is defined by Dorothea Orem as a practice, a performance of activities that the individual carries out for their own benefit to maintain life, aiming at their health and well-being^(7)^. Individuals who take care of themselves are the ones who have the capacity and power to carry out this action. By placing nursing in a position of responsibility for caring, these professionals are called by Orem “self-care agents” who also have powers and capabilities to provide assistance and encourage self-care^(7)^.

Clinical practice and the literature show that self-care for women and men with UI and HTLV 1 has peculiarities, since several health demands, such as difficulty walking, myalgia, loss of sensitivity, sexual, intestinal and skin changes, are associated with urinary complaints. Therefore, self-care measures and interventions in self-care deficit, well-structured for this clientele, contribute to adherence to nursing actions^(1,7)^. The search for information about the facilities and difficulties encountered by women and men in promoting self-care is essential to identify which aspects of nursing care can be improved and what guidelines are necessary to implement more qualified assistance. The feedback of those who suffer and experience the disease, who learn to deal with chronicity and who seek health services to rehabilitate themselves, are able to report on the treatment, inform the best actions that had an effect on them, and which care and self-care strategies were learned or not^(5,6)^. To understand these strategies, it is necessary to identify the factors influencing them, which are described here as Conditioning Facilitating (FF) and Hindering (HF) Factors. Thus, it is hypothesized that knowing the FF and HF factors contributes to the implementation of specific self-care actions for the difficulties presented, besides strengthening the actions that have been successful with the facilitators. This research problem arose from practical experience with this population and the need to understand self-care actions for planning nurses’ actions, to make them more assertive.

The complexity of caring for women and men with UI living with HTLV 1 requires current studies that present clear, concise, and possible information for nursing practice. Therefore, the construction of a technical-technological product will contribute greatly to nursing care, a subject that is still little explored in this professional category. For this purpose, the researcher’s immersion in the lived and practical reality of the social actresses and actors under study contributes to the identification of their needs that will support technology development^(8)^. This study refers to the first phase of the Praxis Model for Technology Development (PMTD) which seeks awareness of praxis as the first step towards building technology^(8)^. Consequently, the objective of this study is to analyze self-care conditioning factors in women and men with urinary incontinence symptoms living with the Human T-Lymphotropic Virus Type 1.

## METHOD

### Design of Study

Qualitative study, resulting from the first phase, Pragmatic, of the PMTD, which deals with a participatory movement, originating from the practice that was configured in the identification of problems, based on the demands of women and men, the recognition of the collective practice to be employed in the context of self-care in life with UI, the formulation of hypotheses on how to intervene, within the scope of Nursing practice, permeated by the relationship between theoretical support (Self-Care Theory), scientific evidence of UI, and HTLV 1 and clinical practice^(8)^.

It is derived from a doctoral dissertation project in nursing, which will proceed to the other phases of the model for producing a social technology product in nursing and health.

### Local

The study was carried out between June and July 2023 at an association supporting people with the HTLV 1 and 2 viruses in the state of Bahia, Brazil. The association was founded in 2010, consisting of approximately 600 members living with HTLV. The selection of this location is justified by its character of social empowerment through mutual support, stimulation of the feeling of collectivity, a place for exchanging knowledge that favors the literacy of its members and the general public, in addition to giving visibility to the group needs and political actions in favor of their rights. Therefore, it constitutes a space for people who are aware of their social and health needs, with the capacity to contribute to the objective proposed.

### Population and Inclusion Criteria

Seventeen people participated in the study using a convenience sample. The inclusion criteria were: self-identifying as women or men, being an adult, living with HTLV, and presenting a complaint of UI. The exclusion criteria were: pregnant women. Women named themselves after flowers and men after car models.

### Data Collection

The hypothesis was selected through practical experience and the need to understand self-care actions and self-care deficits. The pragmatic interpretation of the hypothesis raised was answered in the investigated scenario, where there was already a rapprochement between the moderator, volunteer nurse, the association, and associates. Furthermore, a collection method was chosen, the focus group, which favored the free but targeted expression of women and men for the proposed objectives.

For the focus group meeting, a list of questions was prepared, previously defined by the research group, which aimed to understand how women and men performed self-care and to detect failures in this self-care, through the self-care deficit, and which factors contributed to or hindered this care, with the position of mediator being maintained, without interfering or influencing the dialogues.

The main researcher became the moderator and had three researchers responsible for recording and describing the group’s individual and collective impressions during the meeting. Two meetings for 12 women and five men, lasting 2 hours and 1 hour, respectively, were organized on different days. The specificities of femininities and masculinities were taken into consideration, given the differences between sexes in the way they take care of themselves and see the world, so that the realities experienced could be valued.

The proposed collection method facilitated the implementation of the PMTD, which advocates an approach to the empirical field for better knowledge, observations, and reflections about the experienced reality to be researched^(8)^, contributing to the construction of the content of a technological product, final outcome of a larger study.

Self-care theory permeated data collection, directing dialogues in light of conditioning factors, requirements for self-care, and care agents.

The research team had expertise in the method and the theme, working in teaching, research and nursing care. A training course was carried out for the team, who signed the confidentiality agreement to enter the field.

The invitation to participate in the research was made during face-to-face meetings at the association and through a social networking application managed by members. People willing to participate attended the meetings. They signed the Free and Informed Consent Form for approval and, subsequently, the performance of individual and collective interviews. There were no withdrawals from those who attended the meetings.

An individual data collection instrument was developed, consisting of structured questions regarding social, demographic and clinical conditions, with the aim of understanding the profile of the population under study. The interview lasted 5 minutes and, to capture the responses, the Google Forms® platform was used, with the instrument being applied at the end of the focus group.

### Data Analysis and Treatment

The meetings were audio-recorded on a mobile phone, with an average total time of 3 hours. For content organization and analysis, the speeches were transcribed in the *Microsoft Office Word 2016* and imported into the *Qualitative Data Analysis (webQDA)* software (license granted by the university), as internal sources, creating a corpus that was accessed online, with thorough reading being carried out by other collaborating researchers (pre-analysis)^(9)^.

THE *webQDA* also allowed material exploration, through exhaustive reading, operationalization of the most frequent words in the content of the communications, and their availability in cloud format, a fact that helped the identification of the recording units and the context in which they were used. This allowed free or hierarchical coding (“tree codes”) and the delineation of semantic categories with precision and objectivity^(9)^, according to the second stage of thematic content analysis, proposed by Laurence Bardin^(10)^.

Due to compatibility for operation on various operating systems, interactive layout, easy access, and task sharing^(9)^, the *software webQDA* allowed the researchers and contributors of this study to jointly carry out the final treatment of the results, the inferences, and the interpretation of the findings, submitting it to an internal evaluation of clarity, reflexivity, explanation, and theoretical density.

The codes, categories and textual analysis were influenced by the Theory of Self-Care and Self-Care Deficit and brought the terms closer to the nomenclatures proposed by the Theory^(7)^.

### Ethical Aspects

The project was submitted to the Research Ethics Committee, with opinion number 5.883.662/2023 and in accordance with Resolution 466/2012. The precepts of open science were respected, using the guide *Consolidated Criteria for Reporting Qualitative Research* (COREQ)^(11)^ for qualitative studies, and no artificial intelligence resources were used in the study and writing of this article.

## RESULTS

### The Participants’ Characterization

The interviews consisted of 17 people, 12 women and five men, characterized in epidemiological, social and clinical data, described in Chart 1 below.

**Chart 1:** Characterization of the participants - Salvador, BA, Brazil, 2024.

**Epidemiological data**	**Sex**	12 women, 05 men
	**Age**	Mean age of 51.5 years (35 to 68 years)
	**Color**	Black/brown
**Social data**	**Marital status**	Married and Common law marriage
	**Level of education**	High school and incomplete higher education
	**Religion**	Catholic
	**Family income**	Average of 2 minimum wages
	**Occupation**	Retired and those on leave with social security
**Clinical data**	**Comorbidities**	SH; DM; mental disorders; myelopathy (spastic paraparesis of lower limbs; UI; fecal incontinence; constipation; erectile dysfunction)
	**Time of discovery of the virus**	From 4 to 22 years ago
	**Type of virus transmission**	Sexual and vertical
	**UI Type**	Urgency UI; Stress UI; Incomplete bladder emptying UI
	**UI Complaint Time**	From childhood to 22 years ago
	**Performs catheterization**	Half of the interviewees
	**Use of hydrophilic catheter**	Only 02
	**Complications from using CIC**	Urethritis, UTI

Source: Authors’ elaboration.

In the pragmatic phase, problems in the collective practice of women and men were identified, with the emergence of codes relating theory to practice, bringing emerging needs, which in the processing of the *software* WebQDA are called internal analysis codes, with two being identified: FF with six categories and HF with 08 categories. These codes were classified and analyzed within the Universal Fundamental Requirements, of development and health deviation, described by Dorothea Orem’s Theory^(7)^.

The empirical findings were submitted to the Self-Care Theory in an inductive data abstraction, through concepts, propositions and statements, giving greater methodological/analytical rigor to the study categories^(10)^. The theoretical framework considers the facilities and difficulties for self-care as “basic conditioning factors” that influence, helping or hindering, the ability of self-care. Therefore, for the nurse to define self-care actions, first the internal and external conditioning factors, described in Charts 2 and 3 below, have to be listed.

**Chart 2:** Theoretical framework of the data on the thematic content of women and men living with UI due to HTLV 1, in the exercise of self-care in view of the facilitating conditioning factors for self-care - Salvador, BA, Brazil, 2024.

Fundamental Requirements	Facilitating Conditioning Factors	Thematic Content
Universal requirements	Promotion of religiosity and spirituality	[...] *I thank God for this diagnosis, granted to me, because it was through it that I became stronger in my faith.* (Lily); [...] *I am standing here, glorifying and asking for God’s mercy and getting better every day.”* (Daisy); [...] *I have faith in the orixá and for some reason, I have to go through this.* (Acacia).
Development requirement	Acceptance and adaptation to the new health condition	[...] *When you don’t accept it, it seems like the disease progresses faster in the body.* (Sunflower); [...] *Living with HTLV is also knowing that I have limits and respecting my limits.* (Clatteya); [...] *I can’t go for walks and go where I need to go, but today I deal with it in the best way possible.* (Sandalwood); [...] *I got on the bike, fell and realized that I wouldn’t be able to ride again, but I didn’t give up, I ran after it, I managed to make an adaptation and I started riding again using my hands.* (Maverick).
Development requirement	Search for knowledge about the disease	[...] *One of the things I learned is that we have to know the virus, that helped me.* (Daisy); [...] *When you live with other people who have the virus, it helps you understand and learn about the problem, it is a learning experience.* (Sunflower); [...] *I’m always watching, so as not to be caught off guard. Men don’t want to take care of themselves, but after the virus I take care of myself.* (Beetle).
Health diversion requirement	Promoting the visibility of disabilities, promotes a greater social support network	[...] *We arrive at places, in wheelchairs, sometimes I don’t even open my mouth and people ask: do you need help?* (Cactus); [...] *Now I’m dragging myself along, using my crutch and I tell everyone, I don’t hide anything, consequently, I have a lot of help from my neighbors!* (Orchid).
Universal requirements	Establishment of a formal (association) and informal (family and neighborhood) support network	[...] *I am grateful to have met the association, which lifts me up a lot.* (Gladiolus); [...] *Do you know who helps me a lot to cope? My neighbors.* (Orchid); [...] *My wife’s strength helped me overcome.* (Maverik); [...] *I have a support network, I have 3 daughters and there is one I can count on the most.* (Sandero); *I used to take care of myself, I lived alone, but now my son is living with me, but I always counted on the neighbors who always came to my house.* (Passat).
Health diversion requirement	Search for access to specialized services and professionals	[...] *the nursing network is a very… constructive support.* (Sandalwood); [...] *The professionals put you in the room, talk, so it is a humanized service.* (Sunflower); [...] *The doctor comes in, sees you, writes the prescription and leaves, so the one who stays with you is the nurse and they are the ones who take care of you, right?* (Lavender); [...] *In terms of nursing, I can say that I think the work is perfect, because they are the ones who see the first needs and provide the first care.* (Passat).

Source: Authors’ elaboration.

**Chart 3:** Theoretical framework of the data on the thematic content of women and men living with HTLV 1, in the exercise of self-care in view of the hindering conditioning factors for self-care - Salvador, BA, Brazil, 2024.

Fundamental Requirements	Hindering Conditioning factors	Thematic Content
Health diversion requirement	Locomotion difficulty	[...] *and this difficulty in walking? We lose movement and can’t walk.* (Sandalwood); [...] *Guys, I have terrible falls!* I’ve been hurt a lot, I’ve even hit my head. (Orchid); [...] *there are difficulties in walking, difficulties with an overactive bladder that causes us discomfort and limits our ability to go out.* (Passat).
Universal requirement	Lack of structural adaptation of the residence	[...] *we fall, but we get up. I get out of bed so dazed that I miss the bathroom door. (Sandalwood); [...] Guys, I have terrible falls! (Orchid); [...] At night, so as not to get up all the time, I wear diapers. The house has door frames and I’m having trouble climbing them.* (Desert rose).
Health diversion requirement	Frequent coexistence with pain	[...] *I take pain medicine. I wake up in the middle of the night and have to take it.* (Sandalwood); [...] *I feel pain every day, I wake up with pain, I sleep with pain, I take the medicine, some days I take two… (Sunflower); [...] It’s giving me so much pain, it’s so painful that I cry.* (Carnation) [...] *Suddenly a burning sensation started from the waist down*. (Maverick).
Development requirement	(Dis)caring for yourself to care for others	[...] *I have the problem and at the same time I have to help my family.* (Daisy) [...] *my daughter suffered an accident and I went to Rio Grande do Sul, after that I abandoned the treatment.* (Orchid); [...] *I’m going crazy, I have to take care of my children who are small and I can’t think about myself and I’m full of health problems, right now, I’m suspected of having cancer.* (Acacia).
Universal requirement	Weakening of the informal (family) support network	[...] *no one wakes up at night in the family, I fell on the ground, I almost broke my knee and no one woke up to help me.* (Orchid); [...] *If I end up in a wheelchair, who will take care of me? My son will have to live his life.* (Acacia); [...] *I stayed home alone, you know?* (Sunflower).
Health diversion requirement	(Lack of) knowledge and (lack of) preparation of professionals about the disease	[...] *The doctors in the countryside don’t want to see me. I have to come to the capital*. (Lavender); [...] *It’s disconcerting to go to the doctor and tell him and explain what we have. There we see that the professional doesn’t understand anything, if he prescribes a medication, do not even take it, because he doesn’t understand anything. It’s a problem!* (Passat); [...] *When I arrived he asked me what exam was this? I told him: if you don’t know, who knows?* (Sandero); [...] *The advantage we have, when we use the specialized service, is the knowledge of the professionals. In the private clinic they don’t know anything.* (Beetle).
Universal requirements	Lack of supplies and materials for self-catheterization	[...] *I do catheterization and sometimes I don’t have lubricant, so I have an inflamed urethra.* (Sandalwood); [...] *I just have to talk about the material that we are going to get from the municipal health department, that we ask for something, they don’t send it and they keep stalling, stalling...* (Maverick).
Health diversion requirement	Psychic suffering	[...] *I believe that everyone ends up in a wheelchair one day. So, this makes me anxious, because I have a problem with depression, you know?* [...] *I also went through a very high level of depression, when I found myself with limitations.* (Sunflower); [...] *I had psychological problems when I found out.* (Uno).

Source: Authors’ elaboration.

The “internal and external conditioning” factors are within the scope of the “fundamental self-care requirements”, which are classified by Orem as necessary requirements to be raised for planning care actions. These requirements are divided into: “universal self-care requirements”, known as activities of daily living, balance between basic physiological, psychic, social and emotional functions, necessary for human functioning; “developmental self-care requirements”, which refer to the life cycle, the process of human growth and development, respecting children and the older people with the limitations of age for self-care, as well as the ability to adapt to a new reality of life; and finally, the third, “health deviation self-care requirements,” which are concerned with disease conditions, consequences of a diagnosis, or refer to the absence of care.

By listing the requirements, the nurse understands why some women and men are able to develop their “care agency” and others are not, thus moving towards a self-care deficit.

### Thematic Category 1: Facilitating Conditioning Factors for Promoting Self-Care

This category presents the theoretical framework regarding the FF experienced by women and men with HTLV 1 in the exercise of self-care for the management of UI.

The FF for performing self-care actions were related to strengthening beliefs, through the promotion of spirituality and adherence to religiosity, acceptance and adaptation to the new health condition after the disease caused by HTLV 1. In terms of adherence to recommended therapeutic measures, knowledge related to the health-disease process and self-care practices strengthened the acceptance requirements. Furthermore, other FFs were present in the experiences of women and men, such as the search for promoting visibility of the identity of people with disabilities, with the consequent reach of a social support network, and the establishment of links to constitute this network, enhanced by the associative movement and family relationships and relationships with people in the social circle. Furthermore, the search for access to health services and meeting with specialist professionals for care directed towards clinical management of UI due to HTLV are present in Chart 2.

### Thematic Category 2: Hindering Conditioning Factors for Promoting Self-Care

HFs for promoting self-care were found in the prerequisites reported by the participants. In managing UI, women and men experienced mobility difficulties, problems adapting to the physical structure of the home environment, living with painful manifestations, and weakening of the family support network, especially for women who saw themselves alone for self-care, in contrast to men who had a female family network by their side, just as they gave up their own care to dedicate themselves to others, which contributed to the presence of psychological suffering, producing impacts on psychological well-being and social relationships. Weaknesses in knowledge about HTLV 1, the performance of health professionals and the lack of essential supplies and materials for performing bladder self-catheterization corroborated the HF, which can be viewed in Chart 3.

### Constituent Elements of the Pragmatic Phase of the Pmtd Model

The recognition of the needs of practice, based on the observed reality of empirical data, with the aim of developing nursing and health care technology, occurred through the pragmatic stage of the PMTD.

By raising the requirements of everyday practices and individual and collective problems, a hypothesis was formulated relating all empirical data to the Theory of Self-Care and Self-Care Deficit, as well as to the scientific evidence^(8)^, described in Chart 4, which represents a synthesis of the scientific knowledge of the study, with the deduction of the proposal of a protocol.

**Chart 4:** Survey of constituent elements for the praxis development of technologies in the context of self-care for women and men living with HTLV 1 in the management of UI - Salvador, Bahia, Brazil, 2024.

Constituent elements of the Pragmatic Phase of Praxis Development of Technologies	Findings found in observed practice
Problem identification	There are conditioning facilitating and hindering factors that influence self-care and nursing actions in the fundamental requirements.
Collective practice	Female and male focus groups meeting and individual interviews.
Hypothesis formulation	Know the conditioning, facilitating and hindering factors that contribute to the implementation of self-care and planning of assertive actions by the nurse.
Relationship between theory and practice	The theory of self-care and self-care deficit support the nurse’s actions that are included in a protocol proposal.

Source: Authors’ elaboration.

The survey of FF and HF, the fundamental health requirements, the best self-care actions and intervention in the self-care deficit, were the product of this first phase and served to subsidize the production of the content of a protocol, demonstrating how important this phase of awareness of practice and praxis is.

Immersion in practice helped to break down the complex reality of nurses knowing how to care for women and men with UI living with HTLV 1, considering the multidimensionalities involved in this care, in addition to the emergence of the theory of self-care as a guide for the construction of this phase of the PMTD.

## DISCUSSION

This study sought to investigate the conditioning factors experienced by women and men with urinary incontinence due to human T-lymphotropic virus type 1 to promote self-care. The prescription of self-care actions and the assessment of self-care deficits are preceded by FF and HF which are requirements for assessing self-care needs. The dimension of basic requirements encompasses universal, developmental, and health deviation requirements, and when these three types of requirements are met, human conditions function within the range of normality^(12)^.

Strengthening belief, through the promotion of spirituality and adherence to religiosity, in people with chronic illness, is used as a coping resource that gives meaning to difficult moments, understanding it as a purpose in life. Religions, believing in the existence of a supernatural force that provides support and sustenance to the person who suffers, encourage resignation, positive thinking that a “Greater Being” intercede for the relief of the problem^(13)^. Spirituality/religiosity also contributes to acceptance and adaptation to living with urine loss, promotes greater tolerance to pain, suffering and the understanding that every difficulty has a necessary purpose for growth and evolution^(13,14)^.

The empowerment of women and men to live with urinary incontinence, improve losses, or even become continent, involves the strategy of self-knowledge of their health problem, the causes and the individual effort employed to replicate, at home, the self-care actions prescribed by the nurse^(12,15)^. In addition to the disease becoming visible to oneself, giving visibility to others, not being ashamed to show one’s limitations, asking for help, and recognizing oneself as a person with a disability, but with the ability of self-care, are necessary requirements for implementing self-care^(6,12)^, as well as recognizing the moment when the support network is necessary to make up for the self-care deficit^(16)^.

The associative movement, implemented in the association of people with HTLV, contributes greatly to strengthening bonds between peers and mutual help, creating a formal support network, which besides embracing also develops political actions and civil-social representation in political and governmental agencies^(16)^. In this regard, the nurse needs to incorporate aspects related to social dimensions into the nursing consultation, gathering information and/or indicating participation in collective support groups, associations of people with similar health/illness conditions.

The informal support network made up of family, friends, and neighbors is the basis for making up for self-care deficits. It must be recognized by the nurse so that, when necessary, it can be searched for. By understanding that a person is a multipersonal unit, who is not alone and should not be alone, their means of interaction and relationships must be taken into consideration when assessing needs for constructing self-care actions and self-care deficits^(7,16)^.

The weakening of the support network for women is related to cultural and gender issues, where care is part of the female universe and, therefore, it is up to them to exercise it^(16,17)^. Therefore, the HF due to the lack of a family support network involves the difficulty in finding people to help with the care of women, who seek formal support networks such as associations, support homes, and specialized services to overcome the self-care deficit. The male role of being cared for permeates an FF, in which the self-care deficit is supplied by the involvement of female caregivers throughout the process of controlling or containing UI.

The HFs for the self-care are seen as an identified problem that, in search for a solution, supports the nurse in defining the fundamental requirements under which the person finds him/herself so that, in this way, he/she can implement actions^(18)^. Although some measures are beyond the professional’s jurisdiction, suggestions and referrals can be made aiming at overcoming the self-care deficit, such as physically restructuring the home environment to eliminate mobility difficulties.

Pain experienced by women and men as a chronic consequence of HTLV complications^(19)^ can be analyzed by the nurse from a conservative perspective, through integrative and complementary health practices, as a therapeutic option in promotion and recovery, emphasizing welcoming listening and interaction with oneself, the environment, and society^(20)^. However, drug measures, with medical assistance, can be implemented while maintaining the leading role of Nursing.

The fragility of the healthcare networks of the Brazilian Public Health System (SUS) for the care of people with HTLV is an obstacle in the management of the virus and its complications, since few Brazilian states have specialized services^(2)^. Invisibility and the lack of public health policies contribute to limited assistance, not covering all the care and self-care needs that these women and men require^(2,21)^. The shortage of devices and materials for self-catheterization is another obstacle, leading to discrepancy between what is recommended by the professional and what is distributed by public services. It is necessary to map the dispensing of these products, which helps in the efficient management of distribution by region, respecting the users’ individual needs^(22)^, reducing anxieties caused by uncertainty about whether or not to obtain the devices.

Given the context and the hindering conditioning factors of psychological suffering presented, perceiving women and men with significant emotional weaknesses that influence family and social coexistence, impacting physical and emotional health, reveals the urgent need for constant psychological monitoring, therapeutic listening actions by the nurse, promoting a positive impact on these people, helping them to develop more favorable and assertive action and reaction strategies, producing positive impacts on psychological well-being, implying adaptations in life to better coexist with the virus^(23,24)^. Moreover, efforts should be made to expand nursing interventions to promote self-care capacity and reduce deficits, as evidenced in other health/disease conditions and life processes^(17,18,19,20,21,22,23,24)^.

Nurses have an innate empowerment that can be used to benefit their actions; thus, knowing the clinical and sociodemographic profile, identifying health and psycho-emotional needs, listening to complaints, limitations, social and family reality and self-care of people, contribute to planning the nurse’s actions.

Thus, the identification of conditioning factors for self-care actions and self-care deficits contributes to clinical practice, expanding scientificity in the nursing practice, contributing to the production of technologies and innovation in nursing.

In this study, there are methodological limitations of a theoretical nature, namely: as it is a qualitative cut of the pragmatic phase, it does not yet allow us to know the practical actions of self-care and self-care deficit, only the survey of the conditioning factors that will positively and/or negatively influence these actions, not contemplating all the dimensions of the Theory of Self-Care Deficit and Systems Theory, advocated by Orem. Furthermore, being a group interview, addressing intimate issues such as UI, diaper use, odors, prejudices and stigmas, contributes to possible inhibitions and/or censorship of the participants, which may have reduced the scope and depth of the data gathered; the use of the same research instrument for women and men may have sublimated gender relational aspects specific to each group and/or failed to consider the specificities of sex/gender in relation to genitourinary dysfunctions.

Studies with a larger sample size, with other methodological approaches to self-care, are necessary to support clinical and scientific nursing practice in relation to the clientele investigated.

## CONCLUSION

The experience of women and men with human T-lymphotropic virus type 1 mobilized the universal requirements of development and health deviation, which are significantly impacted by hindering conditioning factors, resulting from difficult ambulation, home inadequate adjustment, frequent living with pain, (lack of) self-care to care for others, weakening of the informal support network, (lack of) knowledge/(lack of) preparation of professionals about the disease, lack of supplies/materials for self-catheterization and psychological suffering. However, they triggered facilitating conditioning factors that contributed for the ability to care for oneself, through the practice of religiosity/spirituality, acceptance/adaptation of the health condition, knowledge of the disease, visibility of disabilities, formal/informal support network, access to specialized services/professionals. This content contributed to the production of innovative technology in the clinical-care field in Nursing and Health.
