# Supplementary material for: Self-care conditioning factors in women and men with urinary incontinence and Human T-Lymphotropic Virus Type 1
Source: Rev Esc Enferm USP. 2025 Jan 27;58:e20240086. doi: 10.1590/1980-220X-REEUSP-2024-0086en (PMC11773682; doi:10.1590/1980-220X-REEUSP-2024-0086en)
Supplement: Translation

# Translation: Condicionantes do autocuidado em mulheres e homens com incontinência urinária e Vírus Linfotrópico T Tipo 1 Humano

**DOI:** 10.1590/1980-220X-REEUSP-2024-0086pt

**Authors:** Rayssa Fagundes Batista Paranhos, Juliana Bezerra do Amaral, Rose Ana Rios David, Anderson Reis de Sousa, Oscar Javier Vergara Escobar, Tatiane Assone dos Santos, Jeferson Moreira dos Santos, Rafael Costa Fernandes

**Affiliations:** 1 Universidade Federal da Bahia, Escola de Enfermagem, Programa de Pós-Graduação em Enfermagem e Saúde, Salvador, BA, Brasil.; 2 Pontificia Universidad Javeriana Sede Bogotá, Bogotá, Colômbia.; 3 Universidade de São Paulo, Departamento de Medicina Legal, Bioética, Medicina do Trabalho e Medicina Física e Reabilitação, São Paulo, SP, Brasil.; 4 Universidade Federal da Bahia, Escola de Enfermagem, Salvador, BA, Brasil.

**Keywords:** Autocuidado, Saúde da Mulher, Saúde do Homem, Vírus Linfotrópico T Tipo 1 Humano, Incontinência Urinária

## Abstract

**Objetivo:**

Analisar os fatores condicionantes do autocuidado em mulheres e homens com sintomas de incontinência urinária vivendo com o vírus linfotrópico T tipo 1 humano.

**Método:**

Estudo qualitativo, norteado pela fase Pragmática do Modelo Práxico para Desenvolvimento de Tecnologias. Participaram 12 mulheres e cinco homens adultos vivendo com Vírus Linfotrópico T Tipo 1 Humano, residentes na Bahia, Brasil. Aplicou-se um instrumento estruturado e grupo focal para coleta dos dados. O material empírico foi submetido à Análise de Conteúdo Temático, mediante o uso do *software* WebQDA e empregou-se a Teoria do Autocuidado/Déficit de Autocuidado.

**Resultados:**

Os condicionantes facilitadores foram utilizados por mulheres e homens como estratégias para o desempenho do autocuidado e os condicionantes dificultadores impediram ou atrapalharam, mas abriram caminhos para direcionar a atuação da enfermeira para suprir os déficits de autocuidado.

**Conclusão:**

Os requisitos de autocuidado foram impactados pelos fatores condicionantes dificultadores, os facilitadores possibilitaram o autocuidado, oportunizando uma tecnologia em enfermagem/saúde.

## INTRODUÇÃO

O vírus linfotrópico T tipo 1 humano (HTLV 1) é transmitido pela contaminação sanguínea, vertical e sexual. Cerca de 5 a 10% da população mundial possui o vírus, sendo prevalente no Brasil em cerca de 0,5 a 1% da população^(1,2)^, e em alguns estados brasileiros, como a Bahia, esse número chega a 1,76% da população^(1)^. São quatro subtipos e a variedade tipo 1 é a que causa mielopatia, acarretando complicações neurológicas como a Mielopatia Associada ao HTLV 1 (HAM), evoluindo com disfunção neurológica do trato urinário inferior (NDTUI), também conhecida como bexiga neurogênica, podendo apresentar sintomas de incontinência urinária (IU), de bexiga hiperativa, esvaziamento vesical incompleto e retenção urinária, levando à necessidade do cateterismo vesical intermitente limpo^(1,3)^. Esse estudo foca nos sintomas da IU que são amplos e genéricos em pessoas que vivem com HTLV 1, diferenciando-a de outras perdas urinárias, uma vez que pode apresentar diversos sintomas isolados ou associados, dificultando o diagnóstico e manejo adequado, consequentemente necessitando de múltiplas abordagens terapêuticas.

A necessidade constante em saber lidar com o problema de saúde crônico modifica a visão do indivíduo diante de si, exigindo dele ações para o próprio cuidado^(4)^. Nesse sentido, as atividades diárias iniciadas e executadas para si mesmo com a finalidade de manter a vida, a saúde e o bem-estar, perpassa sob a ótica do autocuidado. Essas atividades são apreendidas, conforme o conhecimento cognitivo, crenças, valores, hábitos e práticas exercidas ao longo da vida e que podem ser fortalecidas ou prejudicadas, dependendo das orientações profissionais recebidas e da cultura coletiva na qual a pessoa encontra-se inserida^(4,5 6)^.

O autocuidado é definido por Dorothea Orem como uma prática, um desempenho de atividades que o indivíduo realiza em seu benefício para manter a vida, visando sua saúde e bem-estar^(7)^. O indivíduo que se autocuida é aquele que tem capacidade e poder para desenvolver essa ação. Ao colocar a enfermagem em posição de responsabilidade para o cuidar, essas profissionais são chamadas por Orem de “agentes de cuidado” que possuem também poderes e capacidades para oferecer a assistência e estimular o autocuidado^(7)^.

A prática clínica e a literatura mostram que o autocuidado das mulheres e homens com IU e HTLV 1 tem peculiaridades, uma vez que diversas demandas de saúde, como a dificuldade de deambulação, mialgias, perda da sensibilidade, alterações sexuais, intestinais e dérmicas, estão associadas às queixas urinárias. Sendo assim, medidas de autocuidado e intervenções no déficit do autocuidado, bem estruturadas para essa clientela, contribuem para a adesão às ações de enfermagem^(1,7)^. A busca de informações sobre as facilidades e dificuldades encontradas pelas mulheres e homens para a promoção do autocuidado é fundamental para levantar quais pontos do cuidado da enfermagem podem ser melhorados e quais orientações são necessárias para implementar uma assistência mais qualificada. O *feedback* de quem sofre e vivencia a doença, que aprende a lidar com a cronicidade e que procura o serviço de saúde para se reabilitar, é capaz de relatar sobre o tratamento, informar as melhores ações que surtiram efeito em si, e quais estratégias de cuidado e autocuidado foram apreendidas ou não^(5,6)^. Para o conhecimento destas estratégias, necessita-se levantar quais os fatores que as influenciam, sendo aqui descritos como Condicionantes Facilitadores (CF) e Condicionantes Dificultadores (CD). Desse modo, têm-se como hipótese que conhecer os fatores CF e CD contribui para implementação de ações de autocuidado específicas para as dificuldades apresentadas, além de fortalecer as ações que vêm dando certo com os facilitadores. Este problema de investigação surgiu da vivência prática com essa população e da necessidade de conhecer as ações de autocuidado para o planejamento das ações da enfermeira (o), a fim de torná-las mais assertivas.

A complexidade do cuidar de mulheres e homens com IU vivendo com HTLV 1 requer estudos atuais, que apresentem informações claras, concisas e possíveis para a práxis da enfermeira. Sendo assim, a construção de um produto técnico-tecnológico contribuirá, sobremaneira, para a assistência da enfermagem, conteúdo ainda pouco explorado nessa categoria profissional. Para esse propósito, a imersão da pesquisadora na realidade vivida e prática das atrizes e atores sociais em estudo contribui para o levantamento de suas necessidades que subsidiarão o desenvolvimento da tecnologia^(8)^. Esse estudo refere-se à primeira fase do Modelo Práxico para Desenvolvimento de Tecnologias (MPDT) que busca a consciência da práxis como primeira etapa para construção de uma tecnologia^(8)^. Sendo assim, tem-se como objetivo: analisar os fatores condicionantes do autocuidado em mulheres e homens com sintomas de incontinência urinária vivendo com o vírus linfotrópico T tipo 1 humano.

## MÉTODO

### Tipo de Estudo

Estudo qualitativo, fruto da primeira fase, Pragmática, do MPDT, que trata de um movimento participativo, oriundo da prática que se configurou na identificação dos problemas a partir das demandas das mulheres e dos homens, o reconhecimento da prática coletiva a ser empregada no contexto do autocuidado no viver com IU, a formulação de hipóteses de como intervir, no âmbito da atuação em Enfermagem, permeadas pela relação entre a sustentação teórica (Teoria do Autocuidado), evidências científicas acerca do acometimento pela IU e HTLV 1 e a prática clínica^(8)^.

Derivado de um projeto de tese de doutorado em enfermagem, que dará continuidade às outras fases do modelo para produção de um produto de tecnologia social em enfermagem e saúde.

### Local

A pesquisa foi realizada no período de junho e julho de 2023 em uma associação de apoio às pessoas com o vírus HTLV 1 e 2 no estado da Bahia, Brasil. A associação foi fundada em 2010, constituída por, aproximadamente, 600 associados convivendo com o HTLV. A escolha desse local se justifica pelo seu caráter de empoderamento social por meio de apoio mútuo, estímulo ao sentimento de coletividade, local de trocas de conhecimento que favorece o letramento de seus associados e público em geral, além de dar visibilidade às necessidades do grupo e às ações políticas em prol de seus direitos. Destarte, constitui um espaço de pessoas conhecedoras de suas necessidades sociais e de saúde, com capacidade de contribuir com o objetivo que esse estudo propõe.

### População e Critérios de Inclusão

Participaram do estudo 17 pessoas por amostra de conveniência. Os critérios de inclusão foram: auto identificar-se como mulheres e homens, ser adulto, viver com o HTLV e apresentar queixa de IU. O critério de exclusão foram: mulheres grávidas. As mulheres se autodenominaram com nomes de flores e os homens com nomes de marcas de carros.

### Coleta de Dados

A seleção da hipótese ocorreu por meio da vivência prática e necessidade de conhecer as ações de autocuidado e déficit do autocuidado. A interpretação pragmática da hipótese levantada foi respondida no cenário investigado, onde já havia uma aproximação entre a moderadora, enfermeira voluntária, a associação e associados. Além disso, houve a escolha de um método de coleta, o grupo focal, que favoreceu a expressão livre, porém direcionada, das mulheres e homens para os objetivos propostos.

Para o encontro do grupo focal, foi confeccionado um roteiro de perguntas, previamente definido pelo grupo de pesquisa, que objetivou conhecer como mulheres e homens desempenhavam o autocuidado e detectar as falhas nesse autocuidado, por meio do déficit do autocuidado, e quais os fatores que contribuíram ou dificultaram esse cuidado, tendo a preocupação em manter a posição de mediadora, sem interferir ou influenciar nos diálogos.

A pesquisadora principal tornou-se moderadora e contou com três pesquisadoras responsáveis pelas gravações e descrições das impressões individuais e coletivas do grupo, durante o encontro. Foram organizados dois encontros, em dias diferentes, entre 12 mulheres e cinco homens, com duração de 2 horas e 1 hora, respectivamente. Levaram-se em consideração as especificidades das feminilidades e das masculinidades, em dada diferença entre os gêneros na forma de se autocuidar e de ver o mundo, de maneira que as realidades vivenciadas pudessem ser valorizadas.

O método de coleta proposto facilitou a implementação do MPDT, que preconiza uma aproximação com o campo empírico para melhor conhecimento, observações e reflexões acerca da realidade vivida a ser pesquisada^(8)^, contribuindo para a construção do conteúdo de um produto tecnológico, desfecho final de uma pesquisa maior.

A teoria do autocuidado permeou a coleta de dados, direcionando os diálogos à luz dos fatores condicionantes, dos requisitos para o autocuidado e dos agentes do cuidado.

A equipe de pesquisa possuía expertise no método e na temática, atuando no ensino, pesquisa e assistência de enfermagem. Foi realizado curso de capacitação da equipe que assinou o termo de confidencialidade para adentrar ao campo.

O convite para a pesquisa foi realizado nos encontros presenciais na associação e por meio de aplicativo de rede social, gerenciado pelos associados. Compareceram aos encontros as pessoas dispostas a participar, as quais assinaram o Termo de Consentimento Livre e Esclarecido para a anuência e, posteriormente, a realização das entrevistas individuais e coletivas. Não houve desistência de quem compareceu.

Foi elaborado um instrumento de coleta de dados individual, composto por perguntas estruturadas que diziam respeito às condições sociais, demográficas e clínicas, com o interesse em conhecer o perfil da população em estudo. A entrevista teve duração de 5 minutos e, para a captação das respostas, utilizou-se o apoio da plataforma *Google Forms*®, sendo o instrumento aplicado ao final do grupo focal.

### Análise e Tratamento Dos Dados

Os encontros foram audiogravados em aparelho móvel de telefonia, com tempo médio total de 3 horas. Para organização e análise do conteúdo, as falas foram transcritas em formato *Microsoft Office Word 2016* e importadas para o *software Qualitative Data Analysis (webQDA)* (licença dispensada pela universidade), como fontes internas, criando um corpus que foi acessado de modo *online,* sendo realizada a leitura flutuante pelos demais pesquisadores colaboradores (pré-análise)^(9)^.

O *webQDA* também possibilitou a exploração do material, mediante leitura exaustiva, operacionalização das palavras mais frequentes dos conteúdos das comunicações e disponibilizando-as em formato de nuvem, fato que convergiu para identificação das unidades de registro e do contexto em que estavam empregadas. Isso permitiu a codificação livre ou hierarquizada (“códigos em árvore”) e o delineamento de categorias semânticas com precisão e objetividade^(9)^, conforme a segunda etapa da análise de conteúdo temático, proposto por Laurence Bardin^(10)^.

Em razão da compatibilidade para operacionalização em vários sistemas operativos, *layout* interativo, fácil acesso e partilha de tarefas^(9)^, o *software webQDA* contribuiu para que os pesquisadores e colaboradores deste estudo realizassem em conjunto o tratamento final dos resultados, as inferências e a interpretação dos achados, submetendo-o a uma avaliação interna da clareza, reflexividade, explicação e densidade teórica.

Os códigos, categorias e análise textual sofreram influência da Teoria do Autocuidado e Déficit do Autocuidado, além de aproximar os termos às nomenclaturas proposta pela Teoria^(7)^.

### Aspectos Éticos

O projeto foi submetido ao Comitê de Ética em Pesquisa, sob o número do parecer de 5.883.662/2023 e em conformidade com a Resolução 466/2012. Respeitaram-se os preceitos da ciência aberta, utilizando-se o guia *Consolidated Criteria for Reporting Qualitative Research* (COREQ)^(11)^ para estudos qualitativos, e não foram utilizados recursos de inteligência artificial no estudo e redação deste artigo.

## RESULTADOS

### Caracterização Dos Participantes

As entrevistas foram compostas por 17 pessoas, 12 mulheres e cinco homens, caracterizadas em dados epidemiológicos, sociais e clínicos, descritos no Quadro 1 abaixo.

**Quadro 1:** Caracterização das (os) participantes - Salvador, Bahia, Brasil, 2024.

**Dados epidemiológicos**	**Gênero**	12 mulheres, 05 homens
	**Idade**	Média de 51,5 anos (35 a 68 anos)
	**Cor**	Parda e preta
**Dados sociais**	**Estado civil**	Casados e união estável
	**Escolaridade**	Nível médio e superior incompleto
	**Religião**	Católicos
	**Renda familiar**	Média de 2 salários-mínimos
	**Profissão**	Aposentados e afastados com seguro social
**Dados clínicos**	**Comorbidades**	HAS; DM; transtornos psíquicos; mielopatia (paraparesia espástica de membros inferiores; IU; incontinência fecal; constipação; disfunção erétil)
	**Tempo de descoberta do vírus**	De 4 a 22 anos
	**Tipo de transmissão do vírus**	Sexual e vertical
	**Tipo da IU**	IU por urgência; IU por esforço; IU por esvaziamento vesical incompleto
	**Tempo de queixa da IU**	Desde a infância até 22 anos
	**Realiza cateterismo**	Metade das (os) entrevistadas (os)
	**Uso do cateter hidrofílico**	Apenas 02
	**Complicações pelo uso do CIL**	Uretrites, ITU

Fonte: Elaboração própria.

Na fase pragmática, foram identificados problemas da prática coletiva das mulheres e homens, emergindo códigos que relacionam a teoria à prática, trazendo as necessidades emergentes, que no processamento do *software* WebQDA são chamados de códigos internos de análise, sendo identificados dois: CF com seis categorias e CD com 08 categorias. Tais códigos foram classificados e analisados dentro dos Requisitos Fundamentais universais, de desenvolvimento e de desvio de saúde, descritos pela Teoria de Dorothea Orem^(7)^.

Os achados empíricos foram submetidos à Teoria do Autocuidado de forma indutiva da abstração dos dados, mediante os conceitos, proposições e afirmativas, conferindo maior rigor metodológico/analítico às categorias do estudo^(10)^. O enquadramento teórico considera as facilidades e dificuldades para o autocuidado como “fatores condicionantes básicos” que influenciam, ajudando ou atrapalhando, a capacidade de se autocuidar. Desse modo, para a enfermeira definir as ações de autocuidado, faz-se necessário, primeiramente, elencar os fatores condicionantes internos e externos, descritos nos Quadros 2 e 3 abaixo.

**Quadro 2:** Enquadramento teórico dos dados do conteúdo temático das mulheres e homens vivendo com IU pelo HTLV 1, no exercício do autocuidado diante dos condicionantes facilitadores para o autocuidado - Salvador Bahia, Brasil, 2024.

Requisitos Fundamentais	Condicionantes Facilitadores	Conteúdo Temático
Requisitos Universais	Promoção da religiosidade e espiritualidade	[...] *eu agradeço a Deus esse diagnóstico, a mim concedido, porque foi através dele que me fortaleci na minha fé.* (Lírio); [...] *eu estou aqui de pé, glorificando e pedindo a misericórdia de Deus e ficando boa a cada dia”.* (Margarida); [...] *eu tenho fé no orixá e por algum motivo, tenho que passar por isso.* (Acácia).
Requisito de Desenvolvimento	Aceitação e adaptação à nova condição de saúde	[...] *quando você não aceita, parece que a doença evolui mais rápido no corpo.* (Girassol); [...] *viver com HTLV é também saber que eu tenho limites e respeitar os meus limites.* (Clatteya); [...] *eu não consigo fazer minhas caminhadas e ir ao local que necessito ir, mas hoje eu lido com isso da melhor forma possível.* (Sândalo); [...] *subi na bicicleta, caí e percebi que não daria para voltar a andar, mas não desisti, corri atrás, consegui fazer uma adaptação e voltei a andar utilizando as mãos.* (Maverick).
Requisito de Desenvolvimento	Busca por conhecimento sobre a doença	[...] *uma das coisas que aprendi é que temos que conhecer o vírus, isso me ajudou.* (Margarida); [...] *quando você convive com outras pessoas que têm o vírus, ajuda a entender e conhecer o problema, é um aprendizado.* (Girassol); [...] *estou sempre vigiando, para não ser pego desprevenido. Os homens não querem se cuidar, mas depois do vírus eu me cuido.* (Fusca).
Requisito de Desvio de Saúde	Promoção da visibilidade das deficiências, promove maior rede de suporte social	[...] *a gente chega nos lugares, na cadeira de rodas, às vezes eu não abro nem a boca e o pessoal já pergunta: quer ajuda?* (Cacto); [...] *agora eu estou me arrastando, usando a minha muleta e falo para todo mundo, não escondo nada, consequentemente, tenho muita ajuda dos meus vizinhos!* (Orquídea).
Requisitos Universais	Estabelecimento de rede de apoio formal (associação) e informal (familiar e vizinhança)	[...] *agradeço por ter conhecido a associação, que me levanta muito.* (Palma de Santa Rita); [...] *sabe quem me ajuda muito a enfrentar? meus vizinhos.* (Orquídea); [...] *a força da minha esposa me ajudou a superar.* (Maverik); [...] *tenho uma rede de apoio, tenho 3 filhas e uma é que mais posso contar.* (Sandero); *eu me autocuidava sozinho, morava sozinho, mas agora meu filho está morando comigo, mas sempre contei com os vizinhos que sempre iam em minha casa.* (Passat).
Requisito de Desvio de Saúde	Busca por acesso a serviço e profissionais especializados	[...] *a rede de enfermagem é um apoio muito assim… construtivo.* (Sândalo); [...] *os profissionais botam você na sala, conversam, então, é um atendimento humanizado.* (Girassol); [...] *o médico entra, vê você, faz a receita e vai embora, então quem fica com você é justamente a enfermeira e quem cuida são elas, né?* (Alfazema); [...] *a nível de enfermagem posso falar que eu acho perfeito o trabalho, porque elas é que veem as primeiras necessidades, dão o primeiro atendimento.* (Passat).

Fonte: Elaboração própria.

**Quadro 3:** Enquadramento teórico dos dados do conteúdo temático das mulheres e homens vivendo com o HTLV 1 no exercício do autocuidado diante dos condicionantes dificultadores - Salvador, Bahia, Brasil, 2024.

Requisitos Fundamentais	Condicionantes Dificultadores	Conteúdo Temático
Requisito de desvio de saúde	Dificuldade de locomoção	[...] *e essa dificuldade para caminhar? A gente vai perdendo o movimento e não consegue andar.* (Sândalo); [...] *gente, eu levo cada queda! Já me machuquei muito, já bati até a cabeça.* (Orquídea); [...] *há dificuldades para andar, dificuldades com a bexiga hiperativa que nos traz desconforto e limita-nos a sair.* (Passat).
Requisito universal	Falta de adaptação estrutural da residência	[...] *a gente cai, mas se levanta. Eu saio da cama tão atordoada que eu não acerto a porta do banheiro. Gente, eu levo cada queda!* (Orquídea); [...] à *noite, para não levantar o tempo todo, uso fraldas. A casa tem batentes e eu estou com dificuldade de subi-los.* (Rosa do deserto).
Requisito de desvio de saúde	Convivência frequente com a dor	[...] *eu tomo remédio para dor. Acordo de madrugada e tenho que tomar.* (Sândalo); [...] *sinto dor todos os dias, acordo com dor, durmo com dor, tomo o remédio, tem dia que eu tomo dois…* (Girassol); [...] *está me dando muita dor, é dor de chorar. (Cravo*) [...] *de repente logo começou também uma queimação da cintura para baixo*. (Maverick).
Requisito de desenvolvimento	(Des)cuidar de si para cuidar do outro	[...] *eu tenho o problema e ao mesmo tempo eu tenho que ajudar a minha família.* (Margarida) [...] *minha filha sofreu um acidente e eu fui para o Rio Grande do Sul, depois disso abandonei o tratamento.* (Orquídea); [...] *estou para enlouquecer, tenho que cuidar dos meus filhos que são pequenos e não tenho como pensar em mim e estou cheia de problemas de saúde, agora mesmo, estou com suspeita de câncer.* (Acácia).
Requisito universal	Enfraquecimento da rede de apoio informal (familiar)	[...] *ninguém acorda à noite na família, eu caí no chão, quase quebrei meu joelho e ninguém acordou para me ajudar.* (Orquídea); [...] *se eu chegar a ficar na cadeira de rodas, quem vai cuidar de mim? Meu filho vai ter que viver a vida dele.* (Acácia); [...] *eu ficava em casa sozinha, entendeu?* (Girassol).
Requisito de desvio de saúde	(Des) conhecimento e (des) preparo dos profissionais sobre a doença	[...] *os médicos da cidade do interior não querem me atender. Tenho que vir para a capital*. (Alfazema); [...] é desconcertante chegar ao médico e dizer a ele e explicar o que nós temos. Aí já vemos que o profissional não *entende nada, se ele passar um medicamento, nem toma, porque não entende nada. É problema!* (Passat); [...] *ao chegar ele me perguntou que exame é esse? Eu disse: se você não sabe, imagina eu!* (Sandero); [...] *a vantagem que temos, quando usamos o serviço especializado, é o conhecimento dos profissionais. Na clínica particular eles não sabem não.* (Fusca).
Requisitos universais	Carência de insumos e materiais para o autocateterismo	[...] *faço o cateterismo e algumas vezes não tenho o lubrificante, por isso, tenho uretra inflamada.* (Sândalo); [...] *só tenho mesmo que falar é do material que a gente vai pegar na secretaria municipal de saúde, que a gente pede uma coisa, eles não mandam e ficam enrolando, enrolando...* (Maverick); [...]
Requisito de desvio de saúde	Sofrimento psíquico	[...] *eu acredito que todos vão para a cadeira de rodas, um dia. Então, isso me deixa agoniada, porque estou com problema de depressão, tendeu?* [...] *também passei por um nível de depressão muito grande, quando eu me vi com limitações.* (Girassol); [...] *tive problemas psicológicos quando descobri.* (Uno).

Fonte: Elaboração própria.

Os fatores “condicionantes internos e externos” estão dentro do escopo dos “requisitos de autocuidado fundamentais”, os quais são classificados por Orem como requisitos necessários a serem levantados para o planejamento das ações de cuidado. Esses requisitos são divididos em: “requisitos de autocuidado universais”, conhecidos como atividades da vida diária, equilíbrio entre as funções fisiológicas básicas, psíquicas, sociais e emocionais, necessários para o funcionamento humano; “requisitos de autocuidado de desenvolvimento”, que se referem ao ciclo da vida, ao processo de crescimento e desenvolvimento humano, respeitando a criança e idoso com as limitações da idade para o autocuidado, assim como a capacidade de adaptação a uma nova realidade de vida; e por fim, o terceiro, “requisitos de autocuidado de desvio de saúde”, os quais se preocupam com as condições de doença, consequências de um diagnóstico ou referem-se à ausência de cuidados.

Elencando os requisitos, a enfermeira capta o porquê de algumas mulheres e homens conseguirem desenvolver sua “agência do cuidado” e outras (os) não conseguirem, caminhando, então, para o déficit do autocuidado.

### Categoria Temática 1: Condicionantes Facilitadores Para Promoção do Autocuidado

Esta categoria apresenta o enquadramento teórico acerca dos CF vivenciados por mulheres e homens com o HTLV 1 no exercício do autocuidado para o manejo da IU.

Os CF para o desempenho de ações de autocuidado estiveram relacionados com o fortalecimento da crença, mediante a promoção da espiritualidade e adesão à religiosidade, aceitação e adaptação à nova condição de saúde após o acometimento gerado pelo HTLV 1. Em termos de adesão às medidas terapêuticas recomendadas, o conhecimento relacionado ao processo saúde-doença e às práticas de autocuidado fortaleceram os requisitos de aceitação. Além disso, outros CF mostraram-se presentes na vivência das mulheres e dos homens, tal como a busca pela promoção da visibilidade da identidade da pessoa com deficiência, com consequente alcance de rede de apoio social, e o estabelecimento de vínculos para a constituição de uma rede de apoio, potencializados pelo movimento associativista e a relação familiar e de pessoas do círculo de convivência. Outrossim, a busca por acesso a serviços de saúde e o encontro com profissionais especialistas para o cuidado direcionado para manejo clínico da IU pelo HTLV estão presentes no Quadro 2.

### Categoria Temática 2: Condicionantes Dificultadores Para a Promoção do Autocuidado

Os CD para a promoção do autocuidado foram encontrados nos pré-requisitos relatados pelos participantes. Para o manejo da IU, as mulheres e homens vivenciaram dificuldades de locomoção, problemáticas com a adaptação da estrutura física do ambiente domiciliar, convivência com manifestações dolorosas, enfraquecimento da rede de apoio familiar, principalmente para as mulheres que se viram sozinhas para o autocuidado, contrapondo aos homens que tinham uma rede familiar feminina ao seu lado, assim como elas abdicavam do próprio cuidado para se dedicarem a outrem, o que contribuiu para a presença de sofrimento psíquico, produzindo impactos no bem-estar psicológico e nas relações sociais. Fragilidades no conhecimento sobre o HTLV 1, a atuação dos profissionais de saúde e a carência de insumos e materiais essenciais para a realização do autocateterismo vesical corroboraram os CD, que podem ser visualizados no Quadro 3.

### Elementos Constituintes da Fase Pragmática do Modelo Mpdt

O reconhecimento das necessidades da prática, a partir da realidade observada dos dados empíricos, com fins no desenvolvimento de tecnologia de cuidado em enfermagem e em saúde, ocorreu por meio da etapa pragmática do MPDT.

Ao levantar os requisitos das práticas cotidianas e dos problemas individuais e coletivos, formulou-se hipótese relacionando todos os dados empíricos com a Teoria do Autocuidado e do Déficit do Autocuidado, assim como com as evidências científicas^(8)^, descritos no Quadro 4, que representa uma síntese do conhecimento científico do estudo, tendo como dedução a proposição de um protocolo.

**Quadro 4:** Levantamento de elementos constituintes para o desenvolvimento práxico de tecnologias no contexto do autocuidado de mulheres e homens vivendo com o HTLV 1 no manejo da IU - Salvador, Bahia, Brasil, 2024.

Elementos constituintes da Fase Pragmática de Desenvolvimento Práxico de Tecnologias	Achados encontrados na prática observada
Identificação de problemas	Há fatores condicionantes, facilitadores e dificultadores, que influenciam o autocuidado e as ações de enfermagem nos requisitos fundamentais.
Prática coletiva	Encontro de grupos focais, feminino e masculino, e entrevistas individuais.
Formulação de hipóteses	Conhecer os fatores condicionantes, facilitadores e dificultadores, que contribuem para implementação do autocuidado, e planejamento de ações assertivas da enfermeira.
Relação teoria e prática	A teoria do autocuidado e déficit do autocuidado subsidiam as ações da enfermeira que estão inseridas em uma proposta de protocolo.

Fonte: Elaboração própria.

O levantamento dos CF e CD, os requisitos fundamentais de saúde, as melhores ações de autocuidado e intervenção no déficit de autocuidado, foram o produto dessa primeira fase e serviram para subsidiar a produção do conteúdo de um protocolo, demonstrando o quão importante é essa fase de consciência da prática e da práxis.

A imersão na prática ajudou a desmembrar a realidade complexa que é a enfermeira saber cuidar de mulheres e homens com IU vivendo com HTLV 1, considerando as multidimensionalidades que envolvem esse cuidado, além de emergir a teoria do autocuidado como norteadora da construção dessa fase do MPDT.

## DISCUSSÃO

Este estudo buscou investigar os fatores condicionantes vivenciados por mulheres e homens com incontinência urinária pelo vírus linfotrópico T tipo 1 humano para promoção do autocuidado. A prescrição de ações de autocuidado e levantamento de déficit do autocuidado são antecedidos pelos CF e CD que são requisitos para o levantamento das necessidades de autocuidado. A dimensão dos requisitos básicos engloba os requisitos universais, de desenvolvimento e de desvio de saúde, e quando esses três tipos de requisitos são atendidos, há um funcionamento das condições humanas dentro da faixa de normalidade^(12)^.

O fortalecimento da crença, mediante a promoção da espiritualidade e adesão à religiosidade, em pessoas com adoecimento crônico, é utilizado como recurso de enfrentamento que dá sentido aos momentos difíceis, entendendo como um propósito na vida. As religiões, acreditando na existência de uma força sobrenatural que dá suporte e sustentação à pessoa que sofre, estimulam a resignação, o pensamento positivo que um “Ser Maior” intercede pelo alívio do problema^(13)^. A espiritualidade/religiosidade também contribui para a aceitação e adaptação ao viver perdendo urina, promove mais tolerância à dor, ao sofrimento e ao entendimento que toda dificuldade tem um propósito necessário de crescimento e evolução^(13,14)^.

O empoderamento de mulheres e homens para conviver com a incontinência urinária, melhorar as perdas, ou mesmo tornar-se continente, perpassa pela estratégia de autoconhecimento do seu problema de saúde, das causas e do esforço individual empregado para replicar, em domicílio, as ações de autocuidado prescritas pela enfermeira^(12,15)^. Além de a doença tornar-se visível para si, dar visibilidade para o outro, não ter vergonha de mostrar as limitações, de pedir por ajuda, e de se reconhecer como uma pessoa com deficiência, mas com possibilidades de autocuidar, são requisitos necessários para a implementação do autocuidado^(6,12)^, assim como reconhecimento do momento em que a rede de apoio se faz necessária para suprir o déficit do autocuidado^(16)^.

O movimento associativista, implementado na associação de pessoas com HTLV, contribui sobremaneira para fortalecer os vínculos entre os pares e ajuda mútua, criando uma rede de apoio formal, que além de acolhimento, também desenvolve ações políticas e de representação cível-social nas instâncias políticas e governamentais^(16)^. Neste sentido, a enfermeira necessita incorporar na consulta de enfermagem os aspectos relacionados às dimensões sociais, levantando informações e/ou indicando a participação em grupos de apoio coletivo, associações de pessoas com condições semelhantes de saúde/doença.

A rede de apoio informal constituída por familiares, amigos e vizinhos é a base para suprir os déficits de autocuidado e é fundamental ser reconhecida pela enfermeira para, quando necessário, ser acionada. Ao entender que a pessoa é uma unidade multipessoal, que não é só e nem deve estar sozinha, seu meio de interação e relacionamentos devem ser levados em consideração, quando no momento de levantamento das necessidades para construção das ações de autocuidado e déficit do autocuidado^(7,16)^.

O enfraquecimento da rede de apoio às mulheres relaciona-se com questões culturais e de gênero, onde o cuidado faz parte do universo feminino e, desse modo, cabe a elas exercê-lo^(16,17)^. Sendo assim, o CD por falta de rede de apoio familiar perpassa pela dificuldade em encontrar pessoas para ajudar no cuidado das mulheres, as quais buscam rede de apoio formal como associações, casa de apoio e serviços especializados para suprir o déficit do autocuidado. O papel masculino de ser cuidado perpassa por um CF, no qual o déficit de autocuidado é suprido pelo envolvimento de mulheres cuidadoras em todo o processo de controle ou contenção da IU.

Os CD para o autocuidado são vistos como um problema identificado que, em busca de solução, subsidia a enfermeira para a definição dos requisitos fundamentais sob os quais a pessoa se encontra para que, dessa forma, possa implementar ações^(18)^. Muito embora algumas medidas saiam da alçada profissional, sugestões e encaminhamentos podem ser feitos objetivando suprir o déficit do autocuidado, como reestruturação física do ambiente doméstico para dirimir dificuldades de locomoção.

A dor vivenciada pelas mulheres e homens como consequência crônica das complicações do HTLV^(19)^ podem ser analisadas pela enfermeira na perspectiva conservadora, por meio de práticas integrativas e complementares de saúde, como uma opção terapêutica na promoção e recuperação, enfatizando a escuta acolhedora e a interação consigo mesmo, com o meio ambiente e sociedade^(20)^. Não obstante, medidas medicamentosas, com auxílio médico, podem ser implementadas mantendo o protagonismo da Enfermagem.

A fragilidade nas redes de atenção à saúde do Sistema Único de Saúde (SUS) para o cuidado às pessoas com HTLV é um entrave no manejo do vírus e de suas complicações, uma vez que são poucos estados brasileiros que possuem serviços especializados^(2)^. A invisibilidade e a falta de políticas públicas de saúde contribuem para uma assistência limitada, não englobando todas as necessidades de cuidado e autocuidado que essas mulheres e homens precisam^(2,21)^. A escassez de dispositivos e materiais para o autocateterismo é outro entrave, causando discrepância entre o que é preconizado pelo profissional e o que é distribuído pelos serviços públicos. Faz-se necessário o mapeamento da dispensação desses produtos, que ajuda na gestão eficiente da distribuição por regiões, respeitando a necessidade individual do usuário^(22)^, diminuindo ansiedades causadas pela incerteza em obter ou não os dispositivos.

Diante do contexto e dos condicionantes dificultadores de sofrimento psíquico apresentados, perceber as mulheres e homens com fragilidades emocionais importantes que influenciam na convivência familiar e social, repercutindo na saúde física e emocional, revela que urge a necessidade de acompanhamento psicológico constante, ações de escuta terapêutica da enfermeira, promovendo um impacto positivo nessas pessoas, auxiliando-as em desenvolver estratégias de ações e reações mais favoráveis e assertivas, produzindo impactos positivos no bem-estar psicológico, implicando em adaptações na vida para conviver melhor como vírus^(23,24)^. Além disso, deve-se empreender esforços de ampliação das intervenções de enfermagem para a promoção da capacidade de autocuidado e diminuição dos déficits, como evidenciado em outras condições de saúde/doença e processo vitais^(17,18,19,20,21,22,23,24)^.

A enfermeira possui um empoderamento nato e que pode ser utilizado em prol de suas ações; desse modo, conhecer o perfil clínico e sociodemográfico, levantar as necessidades de saúde, psicoemocional, ouvir as queixas, limitações, realidade social, familiar e sobre autocuidado das pessoas, contribuem para o planejamento das ações da enfermeira.

Assim, o levantamento de fatores condicionantes para ações de autocuidado e déficit de autocuidado reverte-se em contribuição para a prática clínica, ampliando a cientificidade no exercício da enfermagem, contribuindo para a produção de uma tecnologias e inovação em enfermagem.

Neste estudo, há limitações metodológicas de ordem teórica, a saber: por ser um recorte qualitativo da fase pragmática, não permite, ainda, conhecer as ações práticas de autocuidado e déficit do autocuidado, apenas o levantamento dos fatores condicionantes que irão influenciar positivamente e/ou negativamente nessas ações, não contemplando todas as dimensões da Teoria do déficit do Autocuidado e Teoria dos Sistemas, preconizadas por Orem. Ademais, sendo uma entrevista grupal, ao abordar assuntos íntimos como IU, uso de fraldas, odores, preconceitos e estigmas, contribuem para possíveis inibições e/ou censura das (os) participantes, podendo ter diminuído a abrangência e a profundidade dos dados apreendidos; a utilização do mesmo instrumento de investigação para as mulheres e os homens pode ter sublimado aspectos relacionais de gênero específicos para cada grupo e/ou a não contemplação das especificidades de sexo/gênero na relação com as disfunções geniturinárias.

Estudos com maior casuística, com outras abordagens metodológicas sobre o autocuidado, fazem-se necessários para subsidiar a prática clínica e científica da enfermagem diante da clientela investigada.

## CONCLUSÃO

A vivência de mulheres e homens com o vírus linfotrópico T tipo 1 humano mobilizou os requisitos universais de desenvolvimento e desvio de saúde, os quais são significativamente impactados por fatores condicionantes dificultadores, decorrentes da dificuldade de locomoção, inadaptação da residência, convivência frequente com a dor, (des)cuidado de si para cuidar do outro, enfraquecimento da rede de apoio informal, (des)conhecimento/(des)preparo dos profissionais sobre a doença, carência de insumos/materiais para o autocateterismo e sofrimento psíquico. Contudo, deflagraram condicionantes facilitadores que contribuíram para a capacidade de autocuidado, mediante a prática da religiosidade/espiritualidade, aceitação/adaptação da condição de saúde, conhecimento da doença, visibilidade das deficiências, rede de apoio formal/informal, acesso à serviços/profissionais especializados. Tal conteúdo contribuiu para a produção de uma tecnologia com inovação no âmbito clínico-assistencial em Enfermagem e Saúde.
